# Association of the blood urea nitrogen to serum albumin ratio on prognosis in patients with bacterial meningitis: a retrospective cohort study

**DOI:** 10.3389/fcimb.2026.1781379

**Published:** 2026-03-18

**Authors:** Anxin Liang, Yaoyao Zhang, Xiaona Li, Xin Zhang, Xin Guo, Kejian Wu, Wen Jiang, Wen Li

**Affiliations:** 1 Department of Neurology, Xijing Hospital, Fourth Military Medical University, Xi’an, China; 2 Department of Mathematics and Physics, School of Basic Medicine, Fourth Military Medical University, Xi’an, China

**Keywords:** albumin, bacterial meningitis, blood urea nitrogen, BUN/ALB ratio, prognosis

## Abstract

**Introduction:**

Early identification of high-risk patients is crucial for improving outcomes of bacterial meningitis (BM) patients. The blood urea nitrogen to albumin (BUN/ALB) ratio has demonstrated prognostic value in various critical illnesses, but its role in BM remains unestablished.

**Methods:**

This retrospective cohort study included 146 adult patients with microbiologically confirmed BM hospitalized between 2010 and 2025. BUN/ALB ratio within 6 hours of admission was calculated. Based on 3-month post-discharge modified Rankin Scale (mRS) scores, patients were dichotomized into favorable (mRS 0–2) and unfavorable (mRS 3–6) prognostic groups. Multivariable logistic regression was used to examine the association between BUN/ALB ratio and unfavorable outcome, adjusting for relevant confounders. The predictive performance was assessed using receiver operating characteristic curve analysis.

**Results:**

Among included patients, 87 (59.6%) had unfavorable outcomes. The unfavorable prognosis group exhibited significantly higher admission BUN/ALB ratios compared to the favorable group (5.23 vs. 2.86, *P* < 0.001). After adjustment for age, gender, diabetes history, headache, neurological deficits, pathogen type, and mechanical ventilation, patients in the highest BUN/ALB tertile (> 5.13) showed a 20-fold increased risk of unfavorable outcome compared to the lowest tertile (adjusted OR = 20.494, *P* < 0.001). A significant dose-response relationship was observed (*P* for trend <0.001). RCS analysis supported a linear association (*P* for no-linearity=0.1503). The BUN/ALB ratio demonstrated discriminative ability with an area under the curve of 0.8.

**Conclusion:**

Admission BUN/ALB ratio independently predicts 3-month unfavorable neurological outcomes in BM patients. This routinely available, low-cost biomarker may serve as a practical tool for early risk stratification and could guide more intensive monitoring and intervention in high-risk patients.

## Introduction

Bacterial meningitis (BM) is a critical medical emergency involving the central nervous system (CNS) and remains a leading global cause of infection-related mortality and long-term neurological disability (Castelblanco et al., 2014). Its incidence exhibits significant geographical disparity, ranging from approximately 0.9 to 80 cases per 100,000 individuals per year between high- and low-income countries (Hasbun, 2019; van de Beek et al., 2021). Despite advances in antimicrobial therapy and critical care, the clinical outcomes of BM remain suboptimal, with reported in-hospital mortality rates of 10-58% (Hasbun, 2022). Among survivors, up to 24% develop chronic neurological sequelae, such as hearing loss or focal neurological deficits (Hasbun, 2022). The disease continues to impose a substantial burden on patients, families, and society.

Bacteria can break through the blood-brain barrier through hematogenous spread or direct invasion routes. Their surface adhesins (such as PspC of Streptococcus pneumoniae and type IV pili of Neisseria meningitidis) bind to specific receptors on endothelial cells, triggering transcellular or paracellular penetration. After entering the subarachnoid space, the bacteria multiply extensively and release pathogen-associated molecular patterns, activating immune cells to produce inflammatory factor storms and complement reactions, leading to the destruction of the blood-brain barrier, brain edema, cerebrovascular lesions, and neuronal damage, constituting the core pathological process of meningitis (Hasbun, 2022). Early identification of high-risk patients with BM is paramount for implementing timely, personalized interventions to improve outcomes. However, the clinical manifestations and imaging features of BM are often nonspecific, and the absence of distinctive biomarkers further complicates precise diagnosis and prognostic assessment. Current prognostic assessment in BM primarily relies on clinical features, inflammatory markers, and neuroimaging findings. Older age, any neurological complications, and severe mental deterioration at admission were significantly associated with unfavorable outcome (Han et al., 2024; He et al., 2025; Teixeira et al., 2025). However, these indicators are highly subjective, and when there is a significant deterioration in high-risk patients, it often means that the optimal intervention opportunity has been missed. Cerebrospinal fluid (CSF) inflammatory markers such as interleukin-6、procalcitonin (PCT) and nucleotide-binding leucine-rich repeat family pyrin domain containing 1 (NLRP3) (Gong et al., 2021), provide valuable insights into intracranial pathology and prognosis. However, CSF-based diagnostics are inherently limited by the need for lumbar puncture, an invasive procedure that is often contraindicated or deferred in critically ill patients with elevated intracranial pressure, coagulopathy, or hemodynamic instability. Consequently, CSF sampling may be delayed or unfeasible precisely in those patients who are at highest risk. There is thus an urgent clinical need for alternative prognostic tools that circumvent invasive sampling, are easily and rapidly obtainable, and integrate systemic pathophysiological information.

Blood-based biomarkers, given their accessibility and repeatability, offer a promising solution (Deng et al., 2025). In recent years, integrated biomarkers have garnered attention for their ability to reflect multiple pathophysiological dimensions simultaneously. Blood urea nitrogen (BUN) and serum albumin (ALB) are routine laboratory parameters, primarily reflecting renal/metabolic status and nutritional/inflammatory status, respectively (Belinskaia et al., 2021). BUN reflects protein intake, endogenous protein catabolism, fluid balance, hepatic urea synthesis, and fluid/renal status in critically ill patient (Faisst et al., 2010). Similarly, albumin also has various biological effects, including maintenance of osmotic pressure, binding and transport of various drugs, and neutralization of free radicals (Deng et al., 2025). Both are established predictors of poor outcomes in critically ill patients (Magnussen et al., 2016; Makowiecki et al., 2024; Deng et al., 2025). BUN-to-ALB ratio has been validated as a strong predictor of mortality and adverse events in various conditions, including community-acquired pneumonia, sepsis, and heart failure (Wang et al., 2023; Shi et al., 2024; Zhang et al., 2024; Zhang et al., 2024). The underlying rationale is that an elevated BUN/ALB ratio may signal a dual assault of “high metabolic stress” (elevated BUN) and “low physiological reserve” (low ALB), a state that may compromise the body’s ability to withstand the challenges of severe infection.

The occurrence of meningitis is associated with a variety of susceptibility factors, including age, infection environment, immune status, as well as cranial brain trauma or surgery. Recent studies have further suggested that meningitis patients often exhibit significant hypercatabolic phenomena under these pathogenic factors. This process is accompanied by persistent inflammation and immunosuppression, and can progress to a persistent inflammation-immunosuppression-hypercatabolic syndrome (PICS) (Voiriot et al., 2022). We believe that there is a close relationship between meningitis and nutritional metabolism: the high catabolic state not only reflects organ dysfunction, but also is related to the continuous decline in protein/proalbumin synthesis capacity, leading to the patient being in a long-term catabolic imbalance. Therefore, the prognosis of meningitis may be profoundly affected by this metabolic disorder. We therefore hypothesize that the BUN/ALB ratio, as an “integrative indicator” of systemic stress and nutritional-inflammatory status, may have predictive value for the prognosis of BM patients. However, its role in the prognostic assessment of BM remains unexplored. To address this knowledge gap, we conducted a retrospective cohort study aiming to determine the association between the BUN/ALB ratio at admission and the 3-month neurological functional outcomes in patients with BM, and to evaluate its predictive performance.

## Methods

### Study design and ethics

This was a single-center, retrospective, observational cohort study. Consecutive adult patients with a definite diagnosis of BM hospitalized in the Department of Neurology of Xijing Hospital between January 1, 2010, and October 31, 2025 were enrolled. This study was approved by the Ethics Committee of Xijing Hospital (approval no. KY20140916-3).

The diagnostic criteria for BM were as follows: 1) Positive bacterial evidence from cerebrospinal fluid (CSF) culture, metagenomic next-generation sequencing (mNGS), or blood culture; 2) Clinical signs or symptoms suggestive of meningitis, including fever, altered consciousness, seizures, acute hydrocephalus, or meningeal irritation signs; 3) At least one of the following CSF laboratory abnormalities: Elevated CSF white blood cell count (> 0.25×10^9^/L), typically with neutrophil predominance; CSF-to-serum glucose ratio < 0.4; or CSF glucose level < 2.5 mmol/L, in the absence of a simultaneously measured blood glucose level.

All patients were initially treated with empirical intravenous antibiotics immediately upon clinical suspicion of BM. The standard initial regimen was a third-generation cephalosporin (e.g., ceftriaxone) or a fourth-generation cephalosporin (e.g., cefepime) combined with vancomycin. Subsequent antibiotic treatment was adjusted according to the results of pathogen identification and antibiotic susceptibility tests.

### Data collection and variables

Data of enrolled patients on demographics, comorbid conditions, presenting symptoms, laboratory and microbiological results, radiological examination, and neurological and systemic complications during the disease process were collected. The blood indicators in this study were collected from the first blood samples collected within 6 hours after the patients’ admission. Three months after discharge, trained coordinators conducted structured telephone interviews within a ±7-day time window around the discharge date to assess the clinical outcomes of the patients using the modified Rankin Scale (mRS). All data were directly entered into the electronic data collection system to ensure quality. A favorable outcome was defined as an mRS score of 0-2, and an unfavorable outcome as a score of 3-6. In this study, meningitis occurring after head trauma with skull fracture or following a neurosurgical procedure was classified as post-neurosurgical meningitis.

### Statistical analysis

The BUN/ALB ratio was calculated as BUN (mg/dL) divided by ALB (g/dL). Categorical variables are expressed as counts and percentages. Continuous variables are presented as means ± standard deviation (SD) or medians with interquartile range (IQR), based on their distribution. Group comparisons were made using the independent t-test or Mann-Whitney U test for continuous variables, and the Chi-square test or Fisher’s exact test for categorical variables.

To investigate the association between BUN/ALB ratio and prognosis, univariable and multivariable logistic regression analyses were performed. BUN/ALB ratio was entered into the models both as a continuous variable and after categorization into tertiles (T1–T3). Variables with a *P* value < 0.05 in the univariate analysis and those considered clinically significant were included in the multivariable models. Results are reported as odds ratios (OR) with 95% confidence intervals (CI). Collinearity was assessed using variance inflation factors (VIF), with a threshold of < 5 considered acceptable.

The non-linear relationship was evaluated using restricted cubic splines (RCS) with 3 knots specified, implemented in the “rms” package (version 6.2-0) within R software (version 4.2.3). Visualizations were created using the “ggplot2” package (version 3.3.5). The predictive performance of BUN/ALB ratio was evaluated by the receiver operating characteristic (ROC) curve. Subgroup analyses were performed to assess the potential interactions. Cases with missing data on BUN, albumin, or mRS scores were excluded from the analysis. All analyses were performed using SPSS 24.0 software (IBM, Armonk, NY). The figures were drawn with GraphPad Prism 8.0.1 (GraphPad, San Diego, CA). A two-tailed *P* value < 0.05 defined statistical significance for all tests.

## Result

### Baseline characteristics

**Figure 1:**
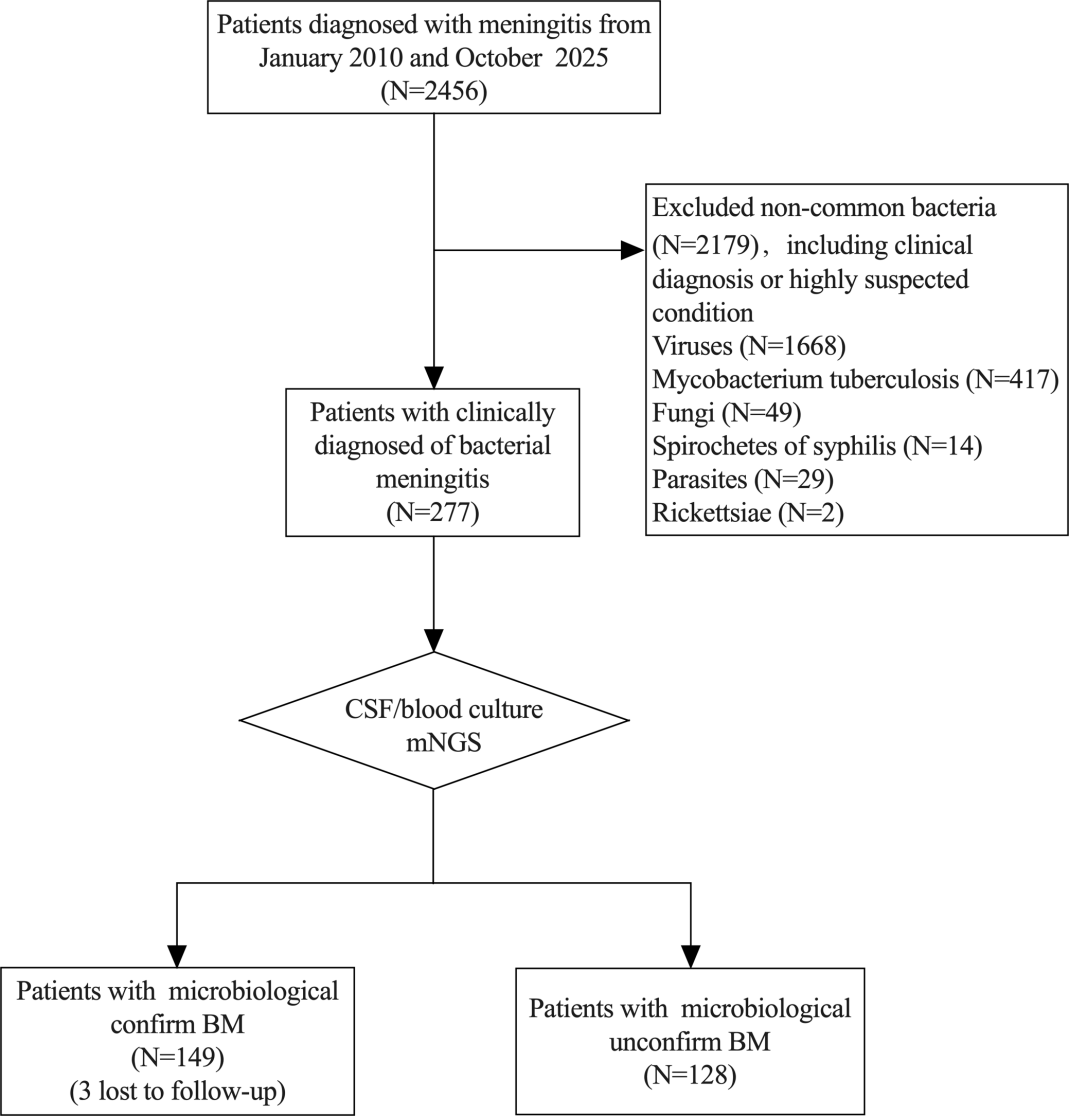
Flowchart of patient enrollment in this study.

A total of 146 patients with etiologically confirmed BM were included in this study, following the exclusion of 3 patients lost to follow-up (**Figure 1**). The cohort comprised 111 males (76%) and 35 females (24%), with a median age of 46.5 (IQR: 33.75, 57.25) years. During the 3-month follow-up, 59 cases (40.4%) had a favorable outcome (mRS 0-2), and 87 cases (59.6%) had an unfavorable outcome (mRS 3-6). Compared to the favorable outcome group, patients in the unfavorable outcome group exhibited the following significant characteristics: In terms of clinical symptoms and complications, the incidence of focal neurological deficits (72.4% vs 45.8%), disorder of consciousness (81.6% vs 33.9%) and cranial imaging abnormalities (86.2% vs 50.8%) was significantly higher (all *P* < 0.001). The incidence of headache (66.7% vs 93.2%) in the unfavorable group is lower. Regarding etiology, patients with an unfavorable prognosis had a higher proportion of Gram-negative bacterial infections (36.8% vs 18.6%) and a lower proportion of Gram-positive bacterial infections (51.7% vs 76.3%) (both *P* < 0.05). In terms of disease severity and treatment indicators, the unfavorable prognosis group had lower Glasgow Coma Scale (GCS) scores (9 vs 15), longer ICU stays (19 vs 13 days), and a higher rate of mechanical ventilation use (39.1% vs 8.5%) (all *P* < 0.001). Additionally, the proportion of patients with a history of diabetes was significantly higher in the unfavorable outcome group (16% vs 2%, *P* = 0.007).

Analysis of key laboratory indicators showed that the unfavorable prognosis group had higher BUN levels (17.64 vs 11.68 mg/dL, *P* < 0.001) and lower ALB levels (3.44 ± 0.61 vs 3.80 ± 0.54 g/dL, *P* < 0.001), resulting in a significantly higher BUN/ALB ratio compared to the favorable outcome group (5.23 vs 2.86 mg/g, *P* < 0.001). There were no significant differences between the two groups in the prevalence of comorbidities such as otitis/sinusitis and chronic renal failure, or in the incidence of common symptoms such as triad of fever, neck stiffness, and change in mental status and fever. The detailed baseline characteristics are shown in **Table 1**.

**Table 1:** Baseline characteristics of patients with favorable and unfavorable outcomes at 3-month follow-up.

Characteristics	Total (n=146)	Unfavorable (n=87)	Favorable (n=59)	*P* value
Age (years)	46.5 (33.75, 57.25)	47 (38, 58)	41 (28, 55)	0.027
Gender (Male n%)	111 (76.0)	69 (79.3)	42 (71.2)	0.259
BMI (kg/m^2^)	22.97 ± 3.71	22.64 ± 3.54	23.45 ± 3.93	0.190
Comorbid conditions (n%)				
Diabetes	18 (12.3)	16 (18.4)	2 (3.4)	0.007
Hypertension	43 (29.5)	31 (35.6)	12 (20.3)	0.047
Cardiovascular disease	6 (4.1)	4 (4.6)	2 (3.4)	0.718
Chronic renal failure	4 (2.7)	2 (2.3)	2 (3.4)	1
Otitis/sinusitis	16 (11)	7 (8)	9 (15.3)	0.171
Postneurosurgical	24 (16.4)	10 (11.5)	14 (23.7)	0.05
Symptoms on presentation (n%)				
Fever	142 (97.3)	85 (97.7)	57 (96.6)	1
Altered mental status	45 (30.8)	22 (25.3)	23 (39)	0.079
Neck stiffness	114 (78.1)	67 (77)	47 (79.7)	0.704
Triad of fever, neck stiffness, and change in mental status	36 (24.7)	17 (19.5)	19 (32.2)	0.082
Headache	113 (77.4)	58 (66.7)	55 (93.2)	<0.001
Nausea and/or vomiting	90 (61.6)	46 (52.9)	44 (74.6)	0.008
Focal neurological deficits	90 (61.6)	63 (72.4)	27 (45.8)	0.001
Characteristics	Total (n=146)	Unfavorable (n=87)	Favorable (n=59)	P value
Disorder of consciousness	91 (62.3)	71 (81.6)	20 (33.9)	<0.001
Seizures	24 (16.4)	18 (20.7)	6 (10.2)	0.092
Cranial imaging abnormality (CT/MRI)	105 (71.9)	75 (86.2)	30 (50.8)	<0.001
Disease severity				
Glasgow Coma Scale score	13 (8, 15)	9 (6, 13)	15 (14, 15)	<0.001
APACHEII score	8 (3, 13)	11 (8, 17)	4 (2, 7)	<0.001
CSF				
Time from onset to LP, days	3 (2, 8.5)	4 (2, 9)	2 (2, 6)	0.085
Leukocyte (×10^6^/L)	647 (194.5, 1840)	604 (268, 1487)	750 (143, 1980)	0.689
Protein (g/L)	2 (1, 3.3)	2.18 (1, 3.85)	1.8 (1.2, 2.8)	0.152
Glucose (mmol/L)	2.1 (1.11, 3.07)	2.1 (1.11, 3.3)	2.01 (1.11, 3.05)	0.791
Chlorides (mmol/L)	114.3 (109.3, 121.05)	112.6 (106.5, 118.9)	117.1 (112.4, 124)	0.003
Serum				
Leukocyte (10^9^/L)	13.02 (8.26, 18.13)	12.72 (8.7, 17.31)	13.69 (7.68, 18.89)	0.766
Creatinine (μmol/L)	76 (58, 91)	75.0 (53, 94)	76.0 (60, 87)	0.735
Urea concentration (mg/dL)	15.29 (10.35, 19.68)	17.64 (11.59, 26.35)	11.68 (8.54, 15.28)	<0.001
Total protein(g/L)	63.35 (58.97, 68.25)	61.4 (57, 66.8)	64.9 (61, 71)	0.001
Albumin (g/dL)	3.62 ± 0.62	3.44 ± 0.61	3.8 ± 0.54	<0.001
CSF/Serum glucose ratio	0.32 (0.17, 0.51)	0.33 (0.15, 0.52)	0.32 (0.20, 0.51)	0.554
BUN/ALB (mg/g)	4.29 (2.64, 5.86)	5.23 (3.45, 8.12)	2.86 (2.22, 3.96)	<0.001
Pathogen (n%)				
Gram-positive bacterium	90 (61.6)	45 (51.7)	45 (76.30)	0.011
Gram-negative bacterium	43 (29.5)	32 (36.8)	11 (18.6)	0.018
Mixed infection	13 (8.9)	10 (11.5)	3 (5.1)	0.182
Therapeutics				
Interval symptoms-antimicrobial therapy>1 day	85 (58.2)	50 (57.5)	35 (59.3)	0.824
Hospitalization duration (days)	23.34 (13,30)	21 (14, 34)	17 (12, 28)	0.061
ICU duration (days)	14.5 (10, 26.25)	19 (11, 30)	13 (8, 17)	0.001
Dexamethasone therapy (n %)	77 (52.7)	48 (55.2)	29 (49.2)	0.475
Mechanical ventilation (n%)	39 (26.7)	34 (39.1)	5 (8.5)	<0.001

### Elevated BUN/ALB ratio as an independent predictor of unfavorable outcome

Logistic regression analyses were conducted to assess the relationship between the BUN/ALB ratio and 3-month functional outcome. In univariable analysis, BUN/ALB ratio as a continuous variable was significantly associated with an increased risk of an unfavorable outcome (OR = 2.00, 95% CI: 1.522, 2.631, *P* < 0.001). This corresponds to approximately 2-fold increase in odds of poor prognosis for each unit increment in the BUN/ALB ratio.

To further investigate this relationship and account for potential confounders, BUN/ALB ratio was subsequently categorized into tertiles (T1: ≤ 2.99, T2: 3.0-5.12, T3: ≥ 5.13) for multivariable analysis. The results are detailed in **Table 2**. After adjusting for age, gender, diabetes (Model 1), patients in the highest tertile (T3) had significantly greater odds of an unfavorable outcome compared to those in the lowest tertile (T1) (adjusted OR = 17.2, 95% CI: 5.119, 57.504, *P* < 0.001). This association remained robust in the fully adjusted model (Model 2), which further included focal neurological deficits, headache, Gram-stain category, and mechanical ventilation. In this final model, the adjusted OR for the T3 group was 20.49 (95% CI: 5.062, 82.867, *P* < 0.001). A significant positive trend was observed across increasing BUN/ALB tertiles (*P* for trend < 0.001), indicating a graded, dose-response relationship between higher BUN/ALB ratios and an elevated risk of poor functional recovery at 3 months.

**Table 2:** Univariable and multivariable logistic regression analyses of the association between BUN/ALB ratio and 3−month unfavorable outcomes.

Character	Crude model		Model 1		Model 2	
	Odd ratios (95% CI)	*P*	Odd ratios (95% CI)	*P*	Odd ratios (95% CI)	*P*
BUN to ALB ratio	2.001(1.522, 2.631)	<0.001	1.977 (1.48, 2.641)	0.000	2.103 (1.448, 3.054)	<0.001
BUN to ALB ratio (in tertiles)						
T1 (≤ 2.99)	1		1		1	
T2(3.0-5.12)	2.128 (0.944, 4.797)	0.069	1.855 (0.774, 4.455)	0.166	1.075 (0.364, 3.171)	0.896
T3(≥ 5.13)	20.706 (6.359, 67.421)	<0.001	17.217 (5.119, 57.504)	<0.001	20.494 (5.062, 82.867)	<0.001
*P* for trend		<0.001		<0.001		<0.001

Crudel model: BUN to ALB ratio; Model 1: Age, gender, diabetes; Model 2: Age, gender, diabetes, headache, focal neurological deficits, Gram stain category, mechanical ventilation.

### Analysis of non-linear relationship

**Figure 2:**
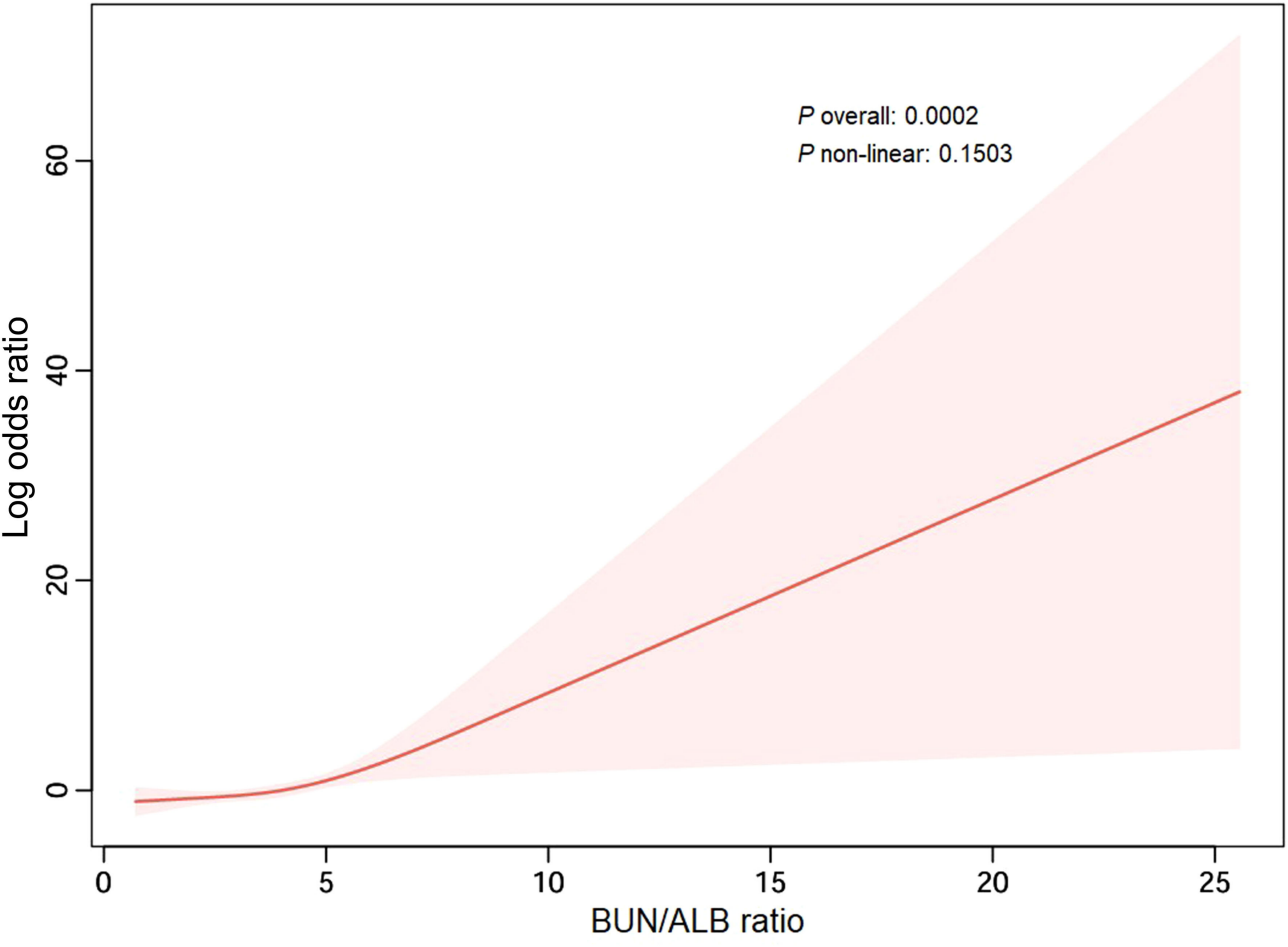
Nonlinear relationship between the BUN/ALB ratio and risk of unfavorable outcome in bacterial meningitis patients. The solid line represents the estimated Log odds ratio (OR) of unfavorable prognosis associated with the BUN/ALB ratio, derived from restricted cubic spline regression. The shaded area indicates the 95% confidence interval.

RCS analysis, adjusted for covariates in Model 2, revealed no significant deviation from linearity (*P* for non-linearity=0.1503), confirming the suitability of BUN/ALB ratio as a continuous linear predictor in our models (**Figure 2**).

### Subgroup analysis and interaction

**Figure 3:**
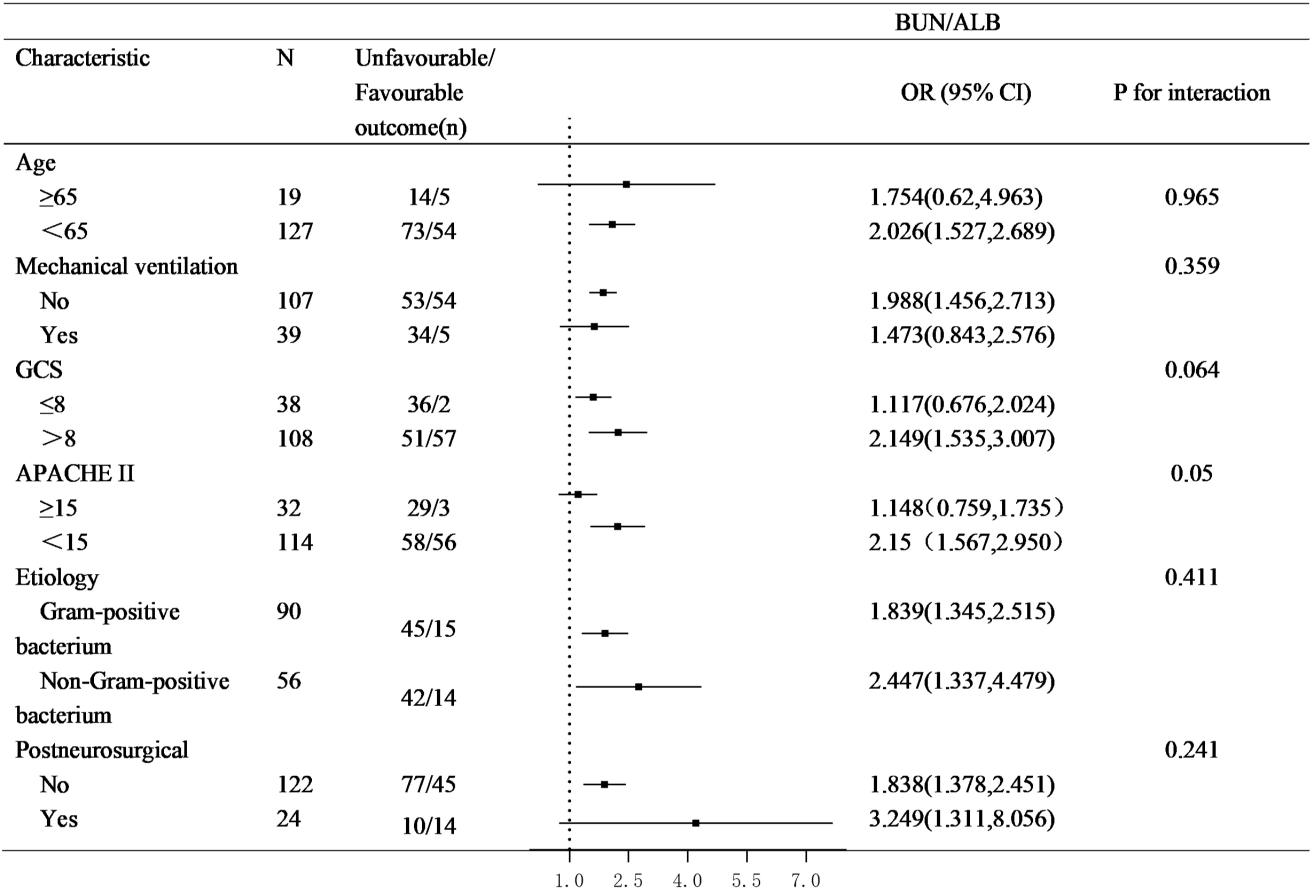
Subgroup analyses of the association between BUN/ALB ratio and unfavorable outcome.

Subgroup analyses were conducted to assess the consistency of the association between high BUN/ALB ratio and poor prognosis across key clinical strata (**Figure 3**). The increased risk associated with high BUN/ALB ratio was consistently observed in subgroups defined by age, mechanical ventilation, history of postneurosurgical, GCS, APACHE II, and gram-stain category. No significant interaction effects were found (all *P* for interaction > 0.05), indicating that the prognostic impact of BUN/ALB ratio was not modified by these variables.

### Predictive performance of BUN/ALB ratio

**Figure 4:**
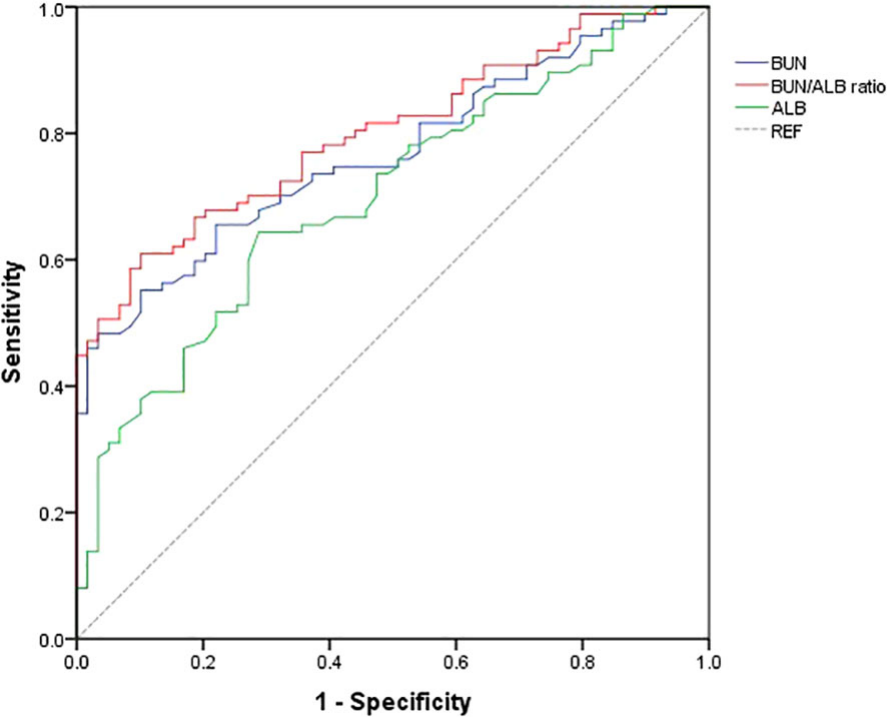
Receiver operating characteristic curve analysis of BUN/ALB ratio for predicting unfavorable prognosis in bacterial meningitis. The area under the curve (AUC) for the BUN/ALB ratio was 0.8 (95% CI: 0.731-0.869).

ROC curve analysis evaluated the predictive accuracy of BUN/ALB ratio for unfavorable 3-month outcomes (**Figure 4**). The area under the curve (AUC) was 0.8 (95% CI: 0.731, 0.869), suggesting a moderate discriminative ability.

## Discussion

This study provides the first systematic evidence that an elevated admission BUN/ALB ratio is an independent predictor of unfavorable short-term neurological outcomes in patients with microbiologically confirmed BM. We found a linear relationship, with the highest ratio tertile associated with a more than twenty fold increased risk, and this association remained robust across key clinical subgroups (AUC = 0.8). The multivariate analysis results of this study strongly support the independence of the BUN/ALB ratio. After sequentially adjusting for demographic characteristics, underlying diseases, and clinical variables reflecting the severity of the disease, the predictive efficacy of this ratio not only did not disappear but actually slightly increased in the fully adjusted model (OR increased from 17.2 to 20.49). This robustness strongly suggests that the BUN/ALB ratio is not merely a simple reflection of the severity of the disease, but may be an integration of multiple key pathogenic mechanisms.

Secondly, compared with the emerging blood biomarkers, the BUN/ALB ratio also demonstrates competitiveness. For instance, the neutrophil-to-lymphocyte ratio (NLR) has been reported to be applicable for predicting the prognosis of central nervous system infections (Kazancioglu et al., 2023). A study showed that its cut-off value for predicting mortality was >8.87 (Baran et al., 2025). PCT outperformed traditional C-reactive protein in differentiating bacterial from viral meningitis (Meini et al., 2025). Additionally, coagulation-related markers such as D-dimer have been proven to predict the adverse outcome of invasive infections caused by Neisseria meningitidis. In this study, the BUN/ALB ratio, after adjusting for infection severity markers (such as mechanical ventilation, Gram staining classification) in multivariate analysis, still maintained a very high effect size (OR > 20), indicating that it may capture a pathological process different from the above indicators - that is, simultaneously reflecting the combined effect of renal perfusion/function status (BUN) and nutritional/systemic inflammatory depletion status (ALB), which may provide more comprehensive prognostic information than single inflammatory or immune markers. Crucially, BUN and ALB are routine, low-cost tests with rapid turnaround, making the BUN/ALB ratio a highly practical and economical composite marker. It integrates information on metabolic stress and nutritional-inflammatory status, emerging as a valuable supplementary tool for early risk stratification in BM management.

The pathophysiology of BM extends beyond the confines of the CNS. Severe intracranial infection can trigger an intense systemic inflammatory response, leading to capillary leakage, relative hypovolemia, a hypercatabolic state, and increased risk of multi-organ dysfunction (Young, 2013; McGill et al., 2016; Galea, 2021). In this context, an elevated BUN/ALB ratio carries significant pathophysiological implications. BUN is the main end-product of protein metabolism in the human body. It can cause immune dysfunction by promoting high catabolism and activating neuro-humoral mechanisms, thereby increasing the mortality risk of critically ill patients with infections and acute kidney injury (Ugajin et al., 2012). However, BUN has low sensitivity and is affected by various factors such as age, high-protein diet, gastrointestinal bleeding, dehydration, and metabolic state. The rise in BUN, beyond reflecting potential prerenal (dehydration, hypoperfusion) or renal factors, may more saliently signify a state of hypercatabolism in critical illness—accelerated protein breakdown leading to increased nitrogenous waste production (Paulus et al., 2025). Conversely, Albumin is synthesized in the liver and has important functions such as maintaining vascular colloid osmotic pressure, maintaining effective circulating blood volume and oxidative-reductive state, and participating in the transport of molecules and drugs (Feng et al., 2019). Current evidence confirms that albumin exerts protective effects on patients through its anti-inflammatory and antioxidant properties. It can promote microcirculation, maintain organ perfusion and function (Ugajin et al., 2012). Decreased serum ALB results from a combination of suppressed hepatic synthesis (negative acute-phase response), increased extravasation due to vascular leakage, and inadequate nutritional intake, directly mirroring the intensity of the inflammatory response and the depletion of the body’s nutritional reserves (Belinskaia et al., 2021; Wiedermann, 2021). Therefore, a high BUN/ALB ratio may delineate a specific “high-depletion” phenotype among BM patients: those enduring the high metabolic burden of infection while lacking sufficient nutritional and physiological reserves to mount an effective response. This compromised state likely impairs neurological repair mechanisms, increases complication risks, and ultimately manifests as worse short-term clinical outcomes. Currently, it has been confirmed that BAR is associated with the risk of death in various diseases such as sepsis, cardiac surgery, pneumonia, and chronic heart failure (Mann and Chung, 2006; Jiang et al., 2019).

Our findings align with research on BUN/ALB ratio in other critical care settings. For instance, an elevated BUN/ALB ratio has been consistently associated with higher mortality in patients with sepsis and community-acquired pneumonia (Ryu et al., 2021; Zou et al., 2021; Wang et al., 2023). This study extends this association to the domain of BM, underscoring the critical role of systemic physiological derangement in determining the final outcome of CNS infections. Notably, the BUN/ALB ratio retained independent predictive value even after adjusting for traditional neurological prognostic factors such as Gram stain category, and focal signs in the multivariable model. This suggests that, in addition to assessing the severity of neurological injury itself, a comprehensive evaluation of the patient’s systemic metabolic and nutritional status is equally vital in prognosticating BM. Although this study attempted to control some clinical variables in the statistical analysis, the adjustments for concurrent clear sepsis, extracranial infection foci, shock, or other organ failure might not be fully adequate. Therefore, the association between a higher BAR and adverse prognosis may be partially mediated by these concurrent systemic critical conditions. Additionally, the incidence of poor prognosis(mRS 3-6) in this study population was approximately 60%, which is similar to the trend observed in previous studies. For instance, a Korean study on meningitis reported that the proportion of patients with mRS scores of 4–6 at 3 months after discharge was approximately 40%, and in our study, this proportion was 39.7%. Additionally, the proportion of patients requiring mechanical ventilation in this study was 39%, which is also roughly equivalent to the proportion (36%) reported in that literature.

From a clinical practice perspective, the BUN/ALB ratio offers distinct advantages: it is derived from routine admission blood tests, incurs no additional cost, and is rapidly available. Our study provides preliminary evidence for its clinical application. For BM patients presenting with a high BUN/ALB ratio (e.g., > 5.13) at admission, clinicians should maintain a high index of suspicion and identify them as a potential high-risk group. This recognition could prompt earlier and more proactive initiation of multimodal intervention strategies.

This study has several limitations. First, as a single-center retrospective observational study, selection bias is inevitable, and external generalizability requires further validation. Second, the sample size is relatively limited, and this limitation is particularly prominent when conducting subgroup analyses, which may affect the statistical power of the subgroup analysis and increase the risk of Type II error. It also reduces the estimation accuracy of effect values, and partially explains the non-significant interaction results. Therefore, the subgroup analysis results in this study should be regarded as preliminary and exploratory, and the conclusions require verification with larger sample sizes. Our study selected patients with a clear etiology, which may be biased. For patients with clinical diagnosis but without pathogen evidence of BM, or patients in regions with significant differences in medical resource levels, the extrapolation of the study conclusions requires caution. Third, we analyzed only a single BUN/ALB ratio measurement at admission and did not track its dynamic trajectory during treatment or its more granular association with outcomes. Fourth, although multiple confounders were adjusted for, the possibility of residual confounding cannot be entirely excluded. Finally, this study focused on validating clinical associations; the specific molecular biological mechanisms through which BUN/ALB ratio influences BM prognosis (e.g., links to specific inflammatory pathways or metabolic reprogramming) were not investigated. To overcome these limitations and more deeply validate the findings of this study, future research should aim to conduct large-scale, multi-center, prospective cohort studies to further explore whether early comprehensive intervention based on the BUN/ALB ratio can truly improve the prognosis of patients with high-risk bacterial meningitis.

## Conclusion

This study confirms that an elevated BUN/ALB ratio at admission is an independent predictor of unfavorable 3-month neurological outcomes in patients with microbiologically confirmed bacterial meningitis. As an easily obtainable composite measure from routine biochemistry, the BUN/ALB ratio provides clinicians with a simple and practical auxiliary tool for the early identification of high-risk BM patients and the implementation of more precise monitoring and supportive care.

## Data Availability

The original contributions presented in the study are included in the article/supplementary material. Further inquiries can be directed to the corresponding authors.
